# Evaluating the tolerability and acceptability of an alcohol-based hand rub – real-life experience with the WHO protocol

**DOI:** 10.1186/s13756-015-0052-9

**Published:** 2015-05-06

**Authors:** Aline Wolfensberger, Nina Durisch, Juliane Mertin, Evelyne Ajdler-Schaeffler, Hugo Sax

**Affiliations:** Division of Infectious Diseases and Hospital Epidemiology, University Hospital Zürich, University of Zürich, Raemistrasse 100, Zurich, CH-8091 Switzerland

**Keywords:** Hand hygiene, Alcohol-based hand rub, Tolerability, Acceptability, World Health Organization

## Abstract

**Background:**

Optimizing user satisfaction with alcohol-based hand rubs (ABHR) may be vital to enhance hand hygiene performance. This study tested the tolerability and acceptability of a new ABHR (EVO9; Ecolab) in healthcare workers under daily working conditions and evaluated the practicability of the corresponding WHO protocol.

**Methods:**

We strictly applied the WHO single product ABHR evaluation protocol. A trained observer assessed hand skin conditions of healthy volunteers using at least 30 ml ABHR per day during their clinical work at baseline, day 3–5 and one month (visit 1–3). Participants rated ABHR tolerability and acceptability at visit 2 and 3. Additionally, we registered study time for participants and study team.

**Results:**

Among 46 volunteers, 76% were female; 37% nurses, 28% physicians. Skin was observer-rated “not” or “incidentally” dry in 64.4%, 77.8%, and 90.9% participants at visit 1, 2, and 3, respectively. EVO9 was scored ≥5 (progressive scale, 1–7) for appearance, intactness, moisture content, and sensation by 95.7%, 97.7%, 88.9%, and 97.8% participants at visit 3, respectively. All WHO benchmarks were exceeded except for “speed of drying” at visit 2, and “texture” at visit 2 and 3. Cumulative study time expenditure was 14 days for the observer and four days for participants.

**Conclusions:**

EVO9 was well tolerated and accepted according to the WHO single ABHR evaluation protocol with the potential for improvement for stickiness. The WHO protocol is feasible but requires considerable time and logistics. It does not preclude bias, in this case especially due to the necessary switch to personal dispensers.

## Background

The World Health Organisation (WHO) ‘Guidelines on Hand Hygiene in Healthcare’ were issued by WHO Patient Safety in May 2009 on the occasion of the launch of the “Save Lives: Clean Your Hands” initiative [1]. Since publication of the guidelines there has been convincing evidence of an improvement in hand hygiene (HH) practices and that, in turn, has resulted in a reduction of hospital acquired infections and/or transmission of multidrug-resistant organisms [2-6]. The WHO suggests five avenues of action as a multimodal strategy of which system change, i.e. facilitated access to well-tolerated alcohol-based handrub (ABHR), is one component.

However, not all initiatives to improve HH are successful and, sustaining high levels of HH compliance among health care personnel are ambitious goals [7,8]. Some of the self-reported reasons given for failing to correctly apply hand cleansing include lack of time, inconveniently located and/or scarcity of facilities, forgetfulness, and agents that cause skin irritations and dryness [9]. In fact, skin tolerability has been reported as one of the main reasons for disinclination towards HH [10-12].

User's acceptance and good skin tolerability are therefore considered being among the most important criteria for the selection of an ABHR [1]. Thus, a product that is pleasant to use with no harmful effect to the hands is a major asset for the promotion of optimal hand hygiene practices.

The WHO provides and promotes the application of two study protocols to evaluate the acceptability and tolerability of an ABHR: “Method 1” for testing a single product planned to be introduced, “Method 2” for comparing two different products [13-15].

The aim of this study was to investigate the tolerability and acceptability of a new ABHR (EVO9; Ecolab) in healthcare workers by using an established WHO protocol, to report the experience with this protocol under real life conditions, and to exemplarily quantify the effort necessary to conduct such a formal evaluation. The study ABHR has successfully been tested according to the European Norm EN1500 and EN12791 and has been registered with Swissmedic under the registration number CHZN3080.

## Methods

### Study setting and protocol

The study was conducted between May and July 2013 at the Division of Infectious Diseases and Infection Control, University Hospital Zurich, Zurich, Switzerland. The methodology was based on the published WHO protocol “Method 1 for Evaluation of tolerability and acceptability of alcohol-based hand rub in use or planned to be introduced” [13]. Three minor modifications were made to the WHO protocol: skin type was assessed with by observer according to Fitzpatrick skin sensitivity type (and not self-assessed by participant), participants were allowed to continue their usual hand care products during the whole study period, and a question on product accessibility was added.

### Study population

The study was carried out in healthy adults of ≥18 years of age. Participants were not allowed to have significant skin or systemic disease or any intolerance to any component of the test preparation. The observer was a medical doctor (AW) trained by an expert who participated in establishing and validating the WHO protocol (MNC, see acknowledgements).

### Study design and endpoints

Each participant received the test product in a 100 ml personal dispenser. Three visits took place: a baseline visit 1 at day 0, visit 2 between day 3 and 5 of product use, visit 3 at the study end after one month of product use.

The quantity of product used was measured at visit 2 and 3 to assure a minimal mean daily use of at least 30 ml of the test product.

The primary endpoint was the observer assessment of skin condition of the participants’ hands using validated scales with higher scores meaning worse conditions including the items *redness* (score, 0–4), *scaliness* (0–3), *fissures* (0–3), and *visual scoring of skin* (1–5). This primary endpoint was evaluated at all three study visits [16].

Secondary endpoints were participant assessment of skin tolerability on a rating scale 1–7 including the items *appearance*, *intactness*, *moisture content*, and *sensation* (all, ‘abnormal’-‘normal’) and *overall skin integrity* (‘very altered’-‘perfect’) and product acceptability including the items *color* (‘unpleasant’-‘pleasant’), *smell* (‘unpleasant’-‘pleasant’), *texture* (‘very sticky’-‘not sticky at all’), *irritation* (‘very irritating’-‘not irritating’), *drying effect* (‘very much’-‘not at all’), *ease of use* (‘very difficult’-‘very easy’), *speed of drying* (‘very slow’-‘very fast’), *application* (‘unpleasant’-‘pleasant’), *overall evaluation* (‘dissatisfied’-‘very satisfied’). Participant evaluations were carried out at visit 2 and visit 3.

### WHO hand rub quality benchmarks

WHO defined benchmark criteria for an acceptable quality for ABHR to help purchasing decision-making. Objective scores for skin condition must score lower than 2 in ≥ 75% of assessments. Participant evaluation of skin condition must score above 4 in ≥ 75% of cases. Participant acceptance must score for *color* and *smell* above 4 in ≥ 50% of the answers and above 4 in ≥ 75% of ratings for the other items in this dimension [13].

### Statistical analysis

The results were interpreted descriptively according to the WHO ‘Method 1’ [13].

### Evaluation of WHO protocol feasibility and applicability

To evaluate the practicability of the WHO protocol, observer and participant time was registered.

The study was formally accepted by the Ethics board of the Canton of Zurich (ID number KEK-ZH-Nr. 2012–0444).

## Results

### Participants

Overall, 46 participants out of a staff of approximately 90 were included after having given their informed consent. Participant mean age was 35 years (standard deviation, ±12); 35 (76%) were female; 17 (37%) were nurses, 13 (28%) medical doctors, five (11%) students, and 11 (24%) belonged to other professions. Fitzpatrick skin sun sensitivity types [17] were: type II, 28 (62%); type III, 14 (31%); and type I, IV, V, one participant (2%) each. Eight participants (17%) were smokers. The remaining participant characteristics are shown in Table 1, data on hand hygiene behavior and product preference in Table 2.

**Table 1:** **Participants characteristics**

**Question**	**Answer**	**Value (N = 46)**
Do you have non work-related activities causing skin damage?*	Yes	12 (26.0%)
Do you normally use of a skin care hand lotion/cream?*	As often as possible	2 (4.2%)
	Several times per day	14 (30.4%)
	Once per day	7 (15.2%)
	Sometimes depending on season	15 (32.6%)
	Rarely	5 (10.9%)
	Never	2 (4.2%)
Do you develop irritative dermatitis?**	Never	31 (67.4%)
	Sometimes depending on season	13 (28.3%)
	Always	0
Do you develop atopic dermatitis?**	Yes	2 (4.2%)
Do you develop allergic rhinitis/allergic conjunctivitis?**	Yes	15 (32.6%)
Are you asthmatic?*	Yes	2 (4.2%)
Do you have Intolerance to alcohol?*	Yes	0
Do you work part time?*	Yes	19 (41.3%)
	Full time equivalent ≤ 50%	4 (8.7%)
For how long have you been using alcohol-based products at work?*	First time	4 (8.7%)
	<1 year	2 (4.2%)
	>1 and < 5 years	6 (13.0%)
	>5 years	33 (71.7%)
Do you think you can improve your own hand hygiene compliance?*	Yes	26 (56.5%)
	Perhaps	10 (21.7%)
It may be difficult to use an alcohol based hand rub because of…	Forgetfulness	Mean, 5.1; median, 6
	Lack of time	Mean, 5.5; median, 6
(Likert scale 1–7; 1, always; 7, never)*	Damaged skin	Mean, 5.4; median, 6
	Poor product accessibility***	Mean, 5.9; median, 6

*One missing answer.

**Two missing answers.

***Question added by the authors outside of the WHO protocol.

**Table 2:** **Hand hygiene behavior and preference of product**

		**Visit 2 (day 3–5)**	**Visit 3 (day 28)**
		**N = 46**	**N = 46**
During how many consecutive days have you used the test product? (days)	3	26 (56.5%)	7 (15.2%)
	4	7 (15.2%)	7 (15.2%)
	5	8 (17.4%)	17 (37.0%)
	6	2 (4.3%)	4 (8.7%)
	7	0	0
	>7	0	10 (21.7%)
	Missing	3 (6.5%)	1 (2.2%)
How often do you have direct contact with patients during your working day? (contacts)	<1	13 (28.3%)	12 (26.1%)
	1 - 5	2 (4.3%)	5 (10.7%)
	6 - 10	6 (13.0%)	6 (13.0%)
	11 - 15	8 (17.4%)	7 (15.2%)
	>15	15 (32.6%)	14 (30.4%)
	Missing	2 (4.3%)	2 (4.3%)
In what percentage of times where hand hygiene is recommended, do you really clean your hands?	≤60%	3 (6.6%)	2 (4.4%)
	70%	7 (15.2%)	3 (6.5%)
	80%	6 (13.0%)	10 (21.7%)
	90%	13 (28.3%)	10 (21.7%)
	100%	16 (34.8%)	18 (39.1%)
	Missing	1 (2.2%)	2 (4.3%)
Has the present study changed your hand hygiene practice?	Yes	17 (37.0%)	17 (37.0%)
	Missing	1 (2.2%)	1 (2.2%)
During your last 5 opportunities for hand hygiene, how many times did you use handrubbing to clean your hands? (times)	≤2	0	1 (2.2%)
	3	6 (13.0%)	4 (8.7%)
	4	12 (26.1%)	11 (24.0%)
	5	26 (56.5%)	29 (63.0%)
	Missing	2 (4.3%)	2 (4.3%)
On average, how often do you practice hand hygiene during a working hour (during the test period)? (times)	<1	2 (4.3%)	2 (4.3%)
	1 – 5	20 (43.5%)	19 (41.3%)
	6 – 10	12 (26.1%)	6 (13.0%)
	11 – 15	6 (13.0%)	9 (20.0%)
	>15	5 (10.7%)	9 (20.0%)
	Missing	1 (2.2%)	1 (2.2%)
Are there differences between the test product and the product used in your hospital? (Likert scale 1 to 7; 1, major difference; 7, no differences)	Mean	3.6	3.3
	Median	3	3
Which product do you prefer?	Usual product	7 (15.2%)	7 (15.2%)
	Test product	26 (56.5%)	30 (65.2%)
	No preference	11 (23.9%)	8 (17.4%)
	Missing	2 (4.3%)	1 (2.2%)
Do you think that the test product could improve your hand hygiene compliance? (Likert scale 1 to 7; 1, yes absolutely; 7, not at all)	Mean	3.6	3.4
	Median	4	3

### Skin tolerability by observer evaluation

Skin condition met the WHO benchmark at all visits and scores improved over time (Table 3).

**Table 3:** **Evaluation of skin condition by trained observer**

**Item**	**Skin condition (score)**	**Visit 1 (day 0)**	**Visit 2 (day 3–5)**	**Visit 3 (day 28)**
Redness	N	46	46	45
	No redness (0)	11 (24%)	16 (35%)	19 (42%)
	Slight redness or blotchy (1)	28 (61%)	25 (54%)	22 (49%)
	Moderate redness (2)	7 (15%)	4 (9%)	4 (9%)
	Strong redness (3)	0	0	0
	Fiery red with oedema (4)	0	1 (2)	0
	Proportion with score < 2*	84.7%	89.1%	91.1%
Scaliness	N	46	46	45
	No scales (0)	29 (63%)	28 (61%)	33 (73%)
	Very slight sporadic scales (1)	13 (28%)	16 (35%)	11 (24%)
	Moderate scales (2)	4 (9%)	2 (4%)	1 (2%)
	Considerable scales (3)	0	0	0
	Proportion with score < 2*	91.3%	95.6%	97.7%
Fissures	N	46	46	44
	No fissures (0)	30 (65%)	34 (74%)	37 (84%)
	Very fine fissures (1)	14 (30%)	12 (26%)	5 (11%)
	Broad, sporadic or several fissures (2)	2 (4%)	0	2 (5%)
	Widespread cracks with haemorrhage or exudate (3)	0	0	0
	Proportion with score < 2*	95.7%	100%	95.7%
Global score	N	45	46	44
	No dry skin or irritations (0)	4 (9%)	8 (17%)	17 (39%)
	Incidental dry skin (1)	25 (56%)	28 (61%)	23 (52%)
	Dry skin and/or redness (2)	13 (29%)	10 (22%)	3 (7%)
	Very dry, whitish rough skin (3)	2 (4%)	0	1 (2%)
	Chappy skin without haemorrhage or exudate (4)	1 (2%)	0	0
	Widespread fissures with haemorrhage or exudate (5)	0	0	0
	Proportion with score < 2*	64.4%	77.8%	90.9%

*WHO benchmark ≥ 75%.

### Skin tolerability by participant evaluation

**Figure 1:**
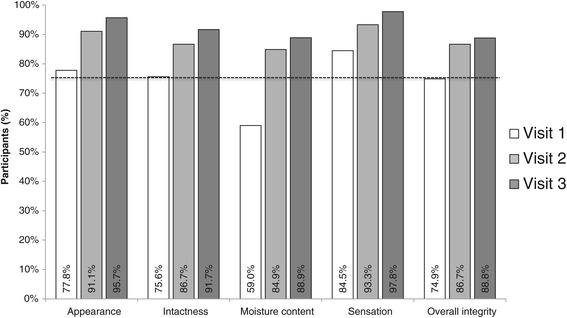
Skin tolerability by participant evaluation. Proportion of participants rating skin tolerability items (Likert scale 1 to 7) > 4; dashed line, WHO minimal pass criteria for alcohol-based hand rub. Visit 1 took place at baseline, visit 2 at day 3–5 and visit 3 at one month of test alcohol-based hand rub use.

The second WHO criterion for skin tolerability by participant rating was also fulfilled at both visit 2 and 3 (Figure 1).

### Product acceptability by participate evaluation

**Figure 2:**
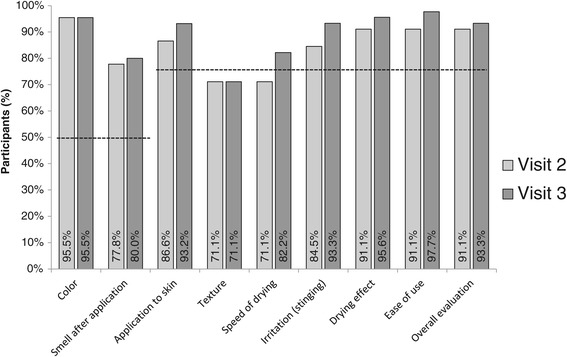
Product acceptability by participant evaluation. Proportion of participants rating skin tolerability items (Likert scale 1 to 7) > 4; dashed line, WHO minimal pass criteria for alcohol-based hand rub. Visit 2 took place at day 3–5 and visit 3 at one month of test alcohol-based hand rub use.

The WHO criteria for product acceptability for the items color and fragrance was fulfilled at each time point. The WHO criteria for all the other items were met in all but two categories at visit 2 and in all but one category at visit 3. The investigational product failed to meet the WHO criterion for acceptability by study participants only for texture at visits 2 and 3 and speed of drying at visit 2. Additionally, while still meeting WHO benchmark ‘smell after application’ received lower scores than the other items (Figure 2).

### Adverse events

One non-serious adverse event (AE) was reported during the study period. The affected participant complained about erythema and pruritus on his/her hands, which was determined to be a local allergic or irritative reaction to the investigational product. The AE was rated as mild, lasted for four hours and disappeared without any treatment. The participant reported similar skin reactions to other products in the past and was withdrawn from the study without further investigation. There were no serious AEs.

### Product consumption

The test ABHR was used at a mean quantity of 35 ml per day at visit 2 and 37 ml per day thereafter. The required frequency of use criterion according to the WHO protocol of a minimum consumption of 30 ml per day was met.

### Practicability assessment

Information, instruction and informed consent procedures required 15 minutes per participant. Time for filling in the questionnaires required five minutes each, time for meeting the observer for evaluation of skin state, distribution and return of bottles accounted for three minutes each. In total every test person spent 40 minutes on study procedures, resulting in 30 hours for all the 46 participants. The observer had to prearrange the study, including preparation of the bottles and questionnaires, which took two working days. Time expenditure for information and recruitment of participants was about two working days. The objective skin evaluation, distribution and return of questionnaires and bottles and measurement of product consumption took about 60 minutes per participant, summing up to six working days in this case. Data analysis and calculation took another four working days. In total the observer/investigator spent 14 working days for conducting the evaluation.

## Discussion

The aim of this study was to investigate the skin tolerability and product acceptability of a new ABHR (EVO9; Ecolab) in healthcare workers under daily working conditions and to evaluate the practicability of the WHO protocol “Method 1 for evaluation of tolerability and acceptability of alcohol-based hand rub in use or planned to be introduced” (WHO 2009). To our knowledge this is the first formal report on the application of the abovementioned WHO protocol.

The primary endpoint concerned participant skin condition rated by a trained observer according to well-established dermatological criteria. The test product surpassed the corresponding WHO benchmark. Moreover, there was a trend to improved ratings with time of use in follow-up visits. These findings were corroborated by participants reporting above-benchmark ratings for “skin tolerability by participant” in every category, i.e., appearance, intactness, moisture content, and sensation, at both study visits. In terms of acceptability the test product failed the WHO benchmark only for texture at visit 2 and 3 and speed of drying at visit 2. After one month of use, 67% of participants preferred the investigational product over the product they normally used.

However, the results have to be interpreted with some caution. Study procedures made it necessary to provide participants with personal bottles of ABHR, which presented a novelty against the habitual bed- and wall-mounted dispensers in our hospital. Many participants told the study observer that they liked the personal ABHR bottles. Such preference might have led to a positive halo effect on product judgment. If feasible, change of provision form for the test product should be avoided since it can lead to positive or negative confounding. For the sake of a real world assessment we allowed participants to continue their habitual hand cream use during the study. This might have offset overall skin condition results favorably but did most likely not change trends over time. Additionally, the quantification in Table 1 that is in accordance with the WHO protocol, allows for comparison with results of other studies. Lastly, it is well known that in an non-blinded study the results tend to be biased towards a beneficial effect of the product under study [18]. This flaw would be mitigated when using the WHO “Method 2” to compare two different products using a randomized blinded protocol.

The WHO protocol proofed to be clear and easy to understand. Yet, its implementation presented some challenges. First, the necessary 14 working days for the observer and four cumulative working days for participants might be perceived as an unusually high workload given the considerable number of potential ABHR available for testing. Thus, if used to select an ABHR for an institution it is advisable to apply this protocol to a limited number of serious ABHR candidates. Second, participants had to use the product for four weeks without interruption for more than five days, otherwise the study duration had to be extended for the same number of days. Fulfilling these requirements in part-time HCWs on irregular shifts proved to be challenging. Third, to prevent inter-individual variation, all skin assessments had to be carried out by the same investigator requiring the person to be available for the whole assessment period.

## Conclusion

The new ABHR (EVO9; Ecolab) was well tolerated and user-accepted with a potential for improvement regarding texture, i.e. stickiness. While the subjective usability and tolerability rating by the users should be interpreted with caution, the skin tolerability assessed by a trained observer may be more reliable. The WHO protocol proofed to be useful but demanding for everyday application.
